# Microbiome Community Structure and Functional Gene Partitioning in Different Micro-Niches Within a Sporocarp-Forming Fungus

**DOI:** 10.3389/fmicb.2021.629352

**Published:** 2021-03-30

**Authors:** Dong Liu, Xinhua He, Caspar C. C. Chater, Jesús Perez-Moreno, Fuqiang Yu

**Affiliations:** ^1^The Germplasm Bank of Wild Species, Yunnan Key Laboratory for Fungal Diversity and Green Development, Kunming Institute of Botany, Chinese Academy of Sciences, Kunming, China; ^2^Department of Land, Air and Water Resources, University of California, Davis, Davis, CA, United States; ^3^Department of Natural Capital and Plant Health, Royal Botanic Gardens, Kew, Richmond, United Kingdom; ^4^Colegio de Postgraduados, Texcoco, Mexico

**Keywords:** GeoChip 5.0, metagenomics, microbial functional genes, Basidiomycota, edible ectomycorrhizal mushrooms, genomic compartmentalization, microbiome

## Abstract

*Thelephora ganbajun* is a wild edible mushroom highly appreciated throughout China. The microbiomes of some fungal sporocarps have been studied, however, their potential functional roles currently remain uncharacterized. Here, functional gene microarrays (GeoChip 5.0) and amplicon sequencing were employed to define the taxonomic and functional attributes within three micro-niches of *T. ganbajun*. The diversity and composition of bacterial taxa and their functional genes differed significantly (*p* < 0.01) among the compartments. Among 31,117 functional genes detected, some were exclusively recorded in one sporocarp compartment: 1,334 genes involved in carbon (*mdh*) and nitrogen fixation (*nifH*) in the context; 524 genes influencing carbon (*apu*) and sulfite reduction (*dsrB*, *dsra*) in the hymenophore; and 255 genes involved in sulfur oxidation (*soxB* and *soxC*) and polyphosphate degradation (*ppx*) in the pileipellis. These results shed light on a previously unknown microbiome and functional gene partitioning in sporome compartments of Basidiomycota. This also has great implications for their potential ecological and biogeochemical functions, demonstrating a higher genomic complexity than previously thought.

## Introduction

Basidiomycota, which includes the mushroom-forming fungi and related taxa, is one of two large divisions within the Fungi Kingdom that, together with Ascomycota, constitute the subkingdom Dikarya (often referred to as “higher fungi”). Over 31,500 species of Basidiomycota have been described (Kirk et al., 2008), which is equal to ∼26% of all known species of Eumycota (Hawksworth and Lücking, 2017). Basidiomycota occurs in all terrestrial ecosystems, and some also live in aquatic habitats. The oldest unambiguous Basidiomycota fossils are from the early Cretaceous (Poinar, 2016), while molecular clock dating suggests that the group may have existed by the upper Devonian, about 340 million years ago (Berbee and Taylor, 1993). Currently, Basidiomycota is a structural and functional component of paramount importance in earth ecosystems as either saprotrophs, pathogens or ectomycorrhizal fungi. They are also culturally significant as food, medicine, drugs and spiritual symbols, with socioeconomic relevance since early human history (Pérez-Moreno et al., 2020). Mushrooms including those from Basidiomycota are currently a main income source for millions of indigenous farmers across the world (FAO, 1997).

*Thelephora ganbajun* M. Zang, a Basidiomycota which belongs to Thelephorales, is an esteemed ectomycorrhizal fungus (EMF) native to China. At present, studies of *T. ganbajun* have focused on its phenolic compounds and polysaccharide contents (Guo et al., 2012), natural antioxidants (Liu et al., 2004; Xu et al., 2016), secondary metabolites (Wen et al., 2016) and bioactive constituents (Cao et al., 2019). So far *T. ganbajun* has never been cultivated due to its uniquely endemic life history (He et al., 2011). Fresh sporocarps of *T. ganbajun* reached up to $120 USD per Kg in 2018 in southwestern China, with an estimated annual retail market of $350 million USD (Pérez-Moreno and Martínez-Reyes, 2014). Due to its high prices, its natural habitats have recently been overexploited, due to an increased demand in domestic and international markets.

*T. ganbajun* establishes ectomycorrhizal symbioses with diverse host trees such as Yunnan pine (*Pinus yunnanensis* Franch.), Khasya pine [*Pinus kesiya* var. *langbianensis* (A.Chev.) Gaussen ex Bui] and Keteleeria (*Keteleeria evelyniana* Mast.) (Mao, 1998; Gui et al., 2005). Such ectomycorrhizal symbioses maintain forest health and functioning (Read and Perez-Moreno, 2003). Previously, the internal transcribed spacer (ITS) variation within *T. ganbajun* was studied in southern China and limited, but detectable, gene flow among species from different provenances has been found (Sha et al., 2008). Furthermore, based on its mitogenome, it has been reported that the evolutionary relationships between this mushroom and other representative taxa of Agaricomycotina are concordant with those found using nuclear genes (Wang et al., 2017). However, studies of the symbiotic relationships of this mushroom species with its associated microbiome and their functional genes are currently lacking.

In particular, “helper bacteria” in the genera *Bacillus, Burkholderia, Pseudomonas* and *Ralstonia* have been shown to facilitate ectomycorrhization by stimulating flavonoid or hormone production, which attracts the mycorrhizal symbiont, alters host innate immune responses, or modifies fungal gene regulation, thereby shifting mycelium from free-living to pre-symbiotic states (Martin et al., 2001; Poole et al., 2001; Kataoka and Futai, 2009; Shinde et al., 2019). The development of ectomycorrhizal fungi is thought to be a microbiome-mediated process, all the way from mycelial growth and ectomycorrhizal formation to the formation of sporomes (Antony-Babu et al., 2014; Li et al., 2018; Baragatti et al., 2019). For Ascomycota EMF species such as truffles (e.g., *Tuber melanosporum* Vittad.), studies have started to dissect the association between bacteria and fungi with sporocarp development, and their roles in aroma formation (Antony-Babu et al., 2014; Vahdatzadeh et al., 2015; Benucci and Bonito, 2016; Splivallo et al., 2019). Sporocarp compartments (such as peridium and gleba) can induce microbial community structure change (Antony-Babu et al., 2014). In contrast, studies related to the Basidiomycota microbiome and functional genes associated to their different compartments are lacking.

**FIGURE 1:**
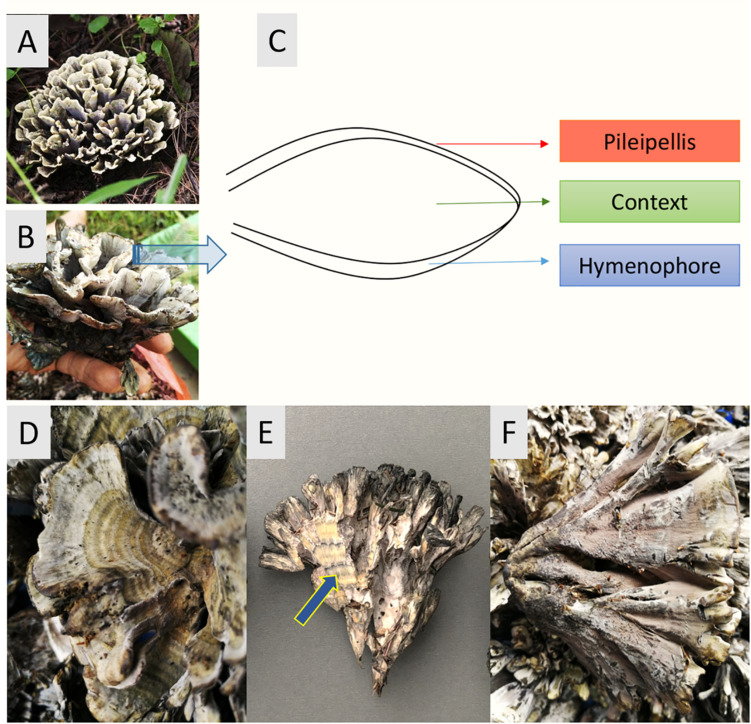
*Thelepora ganbajun* sporocarps and their compartments. **(A,B)** General views of the whole thelephoroid sporocarp growing in *Pinus yunnanensis* forest, its natural habitat. **(C)** Cross section diagram of the three sporocarp compartments. Close-ups of the pileipellis **(D)**, context (blue arrow) showing concentric zones **(E)**, and hymenophore **(F)**.

*T. ganbajun* is characterized by its fleshy base and multiple upward branches. Sub-branches have three layers: (i) an external upper layer called the pileipellis (P); (ii) an inner layer named the context (C) and; (iii) an external lower layer called the hymenophore (H) where the basidiospores are produced (Liu et al., 2006; Mortimer et al., 2012). These unique sporocarp compartments can be considered specific micro-niches for microbes, harboring their own microbiomes and suites of functional genes. From both taxonomic and functional perspectives, this study therefore assessed the composition and abundance of the *T. ganbajun*-associated microbial communities in these three fungal compartments. Our central scientific question was to address whether microbial communities and their potentially active functions could be shaped by the compartments of a sporocarp-forming fungus. We hypothesized that firstly, the compartments of *T. ganbajun* would harbor different microbiome communities (H_1_). Secondly, that the functional gene structure and signal intensities would differ among the compartments (H_2_). Finally, we expected to find higher microbial and functional gene diversities in the context compartment than in both pileipellis and hymenophore (Figure 1), due to the unique microenvironment of this inner compartment (H_3_).

## Materials and Methods

### Sampling Method and Storage

Fresh sporocarps of *T. ganbajun* were collected from a *Pinus yunnanensis* forest in Yunnan province, southwest China (25°8′28′′N, 102°44′17′′E; 1,890 m above sea level), during fruiting season in October 2018. To obtain three independent biological replicates, we harvested ∼3 kg of *T. ganbajun* sporocarps from three different forest sites in the same provenance. In the field, all sporocarps were carefully cut with axenic scalpels to avoid contamination with soil. Field freshly-collected sporocarps were stored in separate plastic bags (40 × 50 cm) which were rolled up instead of being completely sealed in order to avoid anaerobic conditions; and afterward transported on ice to the lab within 2 h. Three sporocarp compartments, the pileipellis, hymenophore and context (Figure 1), from the highest quality fungal material of each biological replicate were cut under axenic conditions into 3–5 slices (>1 cm), and stored in self-sealing bags (60 × 85 mm) ready for DNA extraction. Nine fungal samples were prepared and analyzed in total: three compartments from three biological replicates of sporocarps from three different forest areas.

### DNA Extraction and ITS Identification

Genomic DNA was extracted from 0.3 g of *T. ganbajun* tissues (PowerSoil DNA Isolation Kit, MO BIO, United States) and stored at −20°C for PCR amplification according to Xiong et al. (2016). For bacteria, the 16S rRNA gene’s V4 hypervariable region was amplified by using the 502F and 802R primer pairs, respectively. For fungal communities, we amplified the Internal Transcribed Spacer 2 (ITS2) with the primers ITS3 and ITS4 based on the method of Fujita et al. (2001). Gel-extracted PCR amplicons were purified (EZNA gel extraction kit, Omega, United States) and quantified on a Microplate reader (BioTek, FLx800) using the dsDNA Assay Kit (Invitrogen, P7589). Molecular identification of *T. ganbajun* sporocarps was confirmed through ITS1 analysis using primers ITS5F and ITS1R (Chang et al., 2001). Purified PCR products were sequenced on an ABI 3730XL (Sunny Biotech Company Shanghai, China) and nucleotide sequences were deposited in GenBank (accession numbers MT126627, MT126628, MT126629, and MT126630). Voucher specimens were also deposited at the Herbarium of the Kunming Institute of Botany-Chinese Academy of Sciences (Kunming, China) (accession numbers HSK_111966 and HSKAS_111967).

### Illumina Miseq Sequencing and Data Processing

16S rRNA and ITS libraries were constructed with index codes (NEBNext^®^ Ultra^TM^ DNA Library Prep Kit, Illumina). Library quality was determined using Qubit@ 2.0 Fluorometer (Thermo Fisher Scientific, MA, United States) and Agilent Bioanalyzer 2100 (Agilent Technologies, Waldbron, Germany) before sequencing on an Illumina Hiseq2500 platform to generate 250 bp paired-end reads (Guangdong Magigene Biotechnology Co., Ltd., Guangzhou, China). Sequences were processed through raw read quality control, clean read assembly, and tag quality control. After sequence filter and normalization (28,346 and 28,347 for fungal ITS and bacterial 16S rRNA, respectively), remaining high-quality sequences were classified into operational taxonomic units (OTUs) with 97% similarity using USEARCH OTU clustering^1^ (Edgar, 2010). For bacterial OTUs, UCLUST was used to assign taxonomy against the SILVA database (Quast et al., 2013). For fungal OTUs, BLAST was used to assign taxonomy with the UNITE database (Nilsson et al., 2019). The taxonomic cutoff was set at generic level and the OTUs assigned to same phylum, class, order, family, and genus level were grouped together based on their taxonomic affiliations. Raw sequence data were deposited in the NCBI Sequence Read Archive (accession number PRJNA609601).

### GeoChip Hybridization and Data Processing

Microarray analysis of high quality DNA samples was performed using GeoChip 5.0 as previously described by Feng et al. (2020). Briefly, the Geochip 5.0 functional array covers 570,042 genes including 268,059 functional gene probes from 2,433 gene families related to microbe-driven biogeochemical, ecological, and environmental processes. After purification (QIAquick), DNA samples were labeled with the fluorescent Cy-3 dye and hybridized on a BioMicro platform (MAUI, Salt Lake City, UT, United States) for 5 min, before transfer hybridization on the array surface for 16 h. After microarray scanning, probe signal intensity was calculated using ImaGene 6.0) and spots with signal to noise ratio (SNR) <2 were removed. Probes were considered positive if signals were detected in at least 2/3 of replicate sets. Spot signal intensity was then normalized by relative abundance across samples (the signal intensity of each gene in a sample represents its gene abundance), and gene abundance was log transformed (Cong et al., 2015; Zhang et al., 2017).

### Statistical Analysis

Alpha diversity indices for *T. ganbajun* bacterial community and functional genes were calculated based on normalized bacterial OTUs and normalized functional gene tables, respectively. Specifically, Shannon index considers both number and abundance of OTUs/gene while Simpson index takes into account the number of OTUs/gene, as well as the relative abundance of each OTUs/gene. Dissimilarity of microbial community matrix (beta-diversity) was evaluated using pairwise UniFrac distance and hierarchically clustered via UPGMA (Kahl, 2015). UniFrac distance of the functional gene matrix was visualized using unweighted NMDS. Significant differences of distance matrixes among the three *T. ganbajun* compartments (context = C, hymenophore = H, and pileipellis = P) were simultaneously tested using non-parametric multivariate analysis of variance (adonis), multiple response permutation procedure (MRPP), and the analysis of similarities (ANOSIM) within the Vegan R package (O’Connor, 1988). GeoChip analysis of gene signal intensity between multiple groups (C vs. H; H vs. P; C vs. P) was conducted to identify the top 10 genes that showed the largest significant difference in abundance among compartments. Key functional genes (including main gene category and subcategory) of microbial communities in *T. ganbajun* compartments were also tested by ANOVA. Detrended correspondence analysis was conducted to obtain the length of first axis, RDA (Redundancy analysis) or CCA (Canonical correlation analysis) was chosen based on the value of DCA 1 (>4, CCA; <3 RDA; 3–4 RDA/CCA). Because the length of first axis of DCA was smaller than 3, RDA was chosen to know the relationship between microbial community composition at genus level and functional gene structure. A Monte-Carlo permutation test showed that the top 60 genera from amplicon data were used as vectors, and 16 out of these genera showed a highly significant correlation (*P* < 0.05) with the functional gene structure within the compartments (see Figure 5).

## Results

### Microbiome Diversity

A total of 280,503 16S sequences for bacterial community were obtained from the three *T. ganbajun* compartments. Cleaned reads ranged from 37,195 to 53,982 and were then rarefied to 31,167 sequences per sample. Bacterial sequences within the proteobacteria phylum were the most abundant (62.3–87.6%), followed by Bacteroidetes (10.1–35.3%) and other phyla (1.8–2.3%). As for functional genes, a total of 31,117 genes (29,350 overlapped and 1,767 unique genes) were detected in the compartments, including 26,874 genes from bacteria, 3,300 from fungi, and 943 from archaea.

**FIGURE 2:**
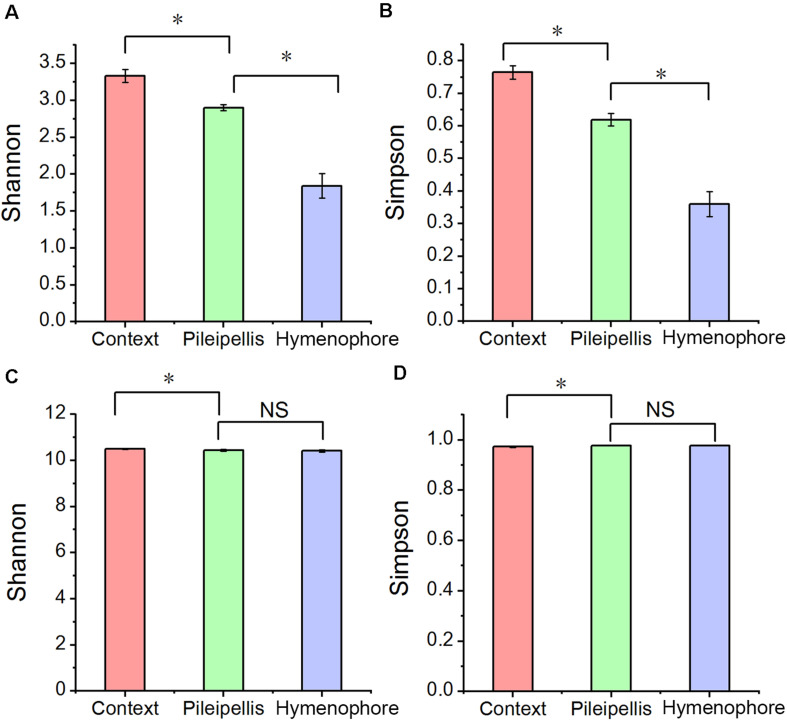
The alpha diversity changes in *Thelephora ganbajun* bacterial communities **(A,B)** and functional genes **(C,D)** in three tissue compartments. For individual figures, significant differences between compartments are indicated with an asterisk (Tukey HSD, *P* < 0.05) and NS is not significant (*P* > 0.05).

Alpha bacterial diversity and overall microbial functional diversity were significantly different (*P* < 0.001) according to Shannon and Simpson indices (Figure 2). The highest bacterial diversity was found in the context (C) and the lowest in the hymenophore (H). In contrast, fungal diversity was not significantly different among the studied compartments (Supplementary Table 1).

**FIGURE 3:**
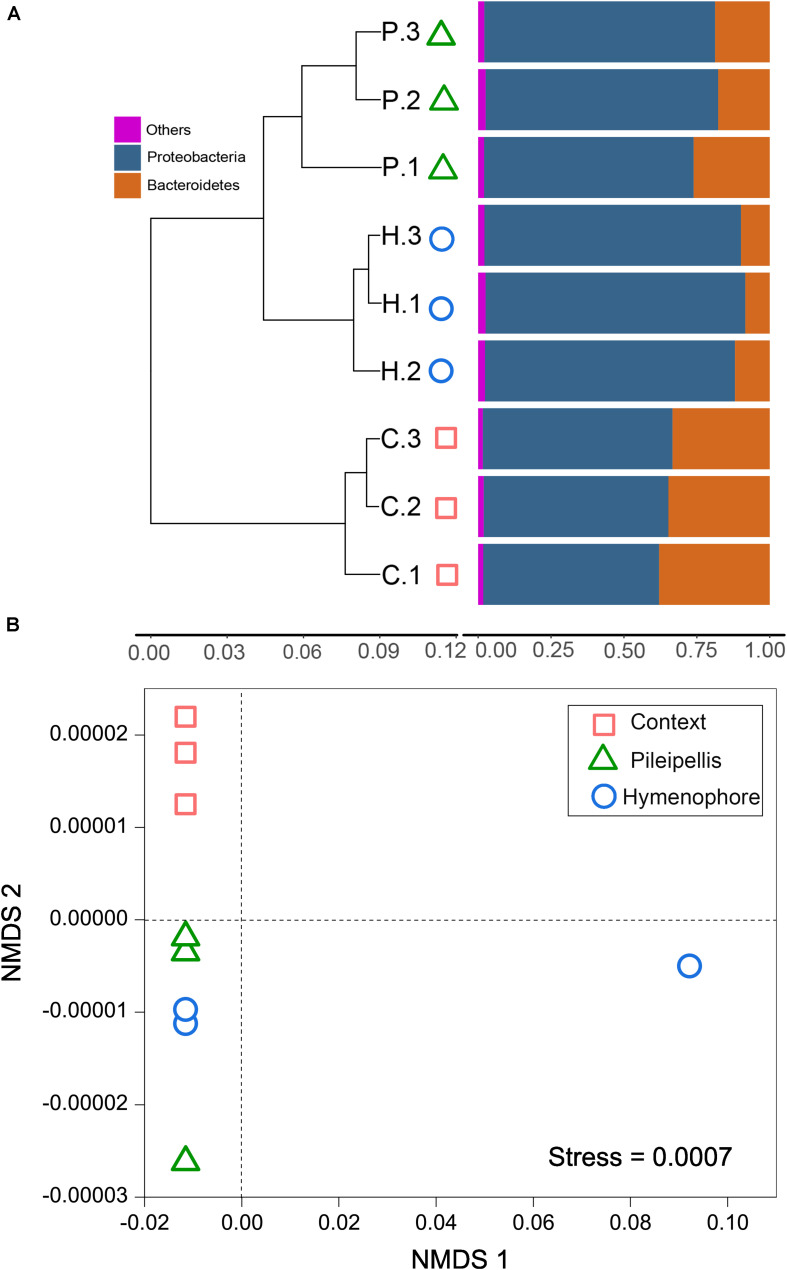
The beta diversity changes in *Thelephora ganbajun* bacterial communities. **(A)** Two-way cluster based on an unweighted UniFrac UPGMA tree; and **(B)** functional genes analyzed via unweighted non-metric multidimensional scaling (NMDS) plots in three sporocarp compartments. In **(A)** abbreviations are C, context; H, hymenophore; P, pileipellis and the numbers 1, 2, 3 denote the replicates for each compartment.

Beta-diversity of both functional genes and bacterial communities differed significantly (*P* < 0.01) among compartments, as revealed by the three (MRPP, ANOSIM, and adonis) non-parametric dissimilarity tests (Table 1). Consistently, cluster and NMDS ordinations indicated similar compartment-based separating trends (Figure 3); the pileipellis and hymenophore clustered tightly compared to the context (Figure 3). In contrast, the context-inhabiting bacterial community was independently clustered and characterized by a higher relative abundance of Bacteroidetes (∼35%), as compared with that of the pileipellis (∼20%) and hymenophore (∼10%) (Figure 3A).

**TABLE 1:** Microbial taxonomic and functional gene composition dissimilarities among three *Thelephora ganbajun* sporocarp compartments (context, hymenophore, and pileipellis).

Statistical approaches	Bacterial	Functional	Fungal
**MRPP^a^**			
Delta	0.764	0.164	−0.113
*p*	**0.004^b^**	**0.004**	0.518
**ANOSIM**			
*R*	1	0.43	−0.029
*p*	**0.006**	**0.003**	0.425
**Adonis**			
*F*	42.838	2.511	0.585
*p*	**0.007**	**0.004**	0.479

*^*a*^MRPP, multi-response permutation procedure.*

*^*b*^Significant effects are shown in bold.*

### Key Function Genes

**FIGURE 4:**
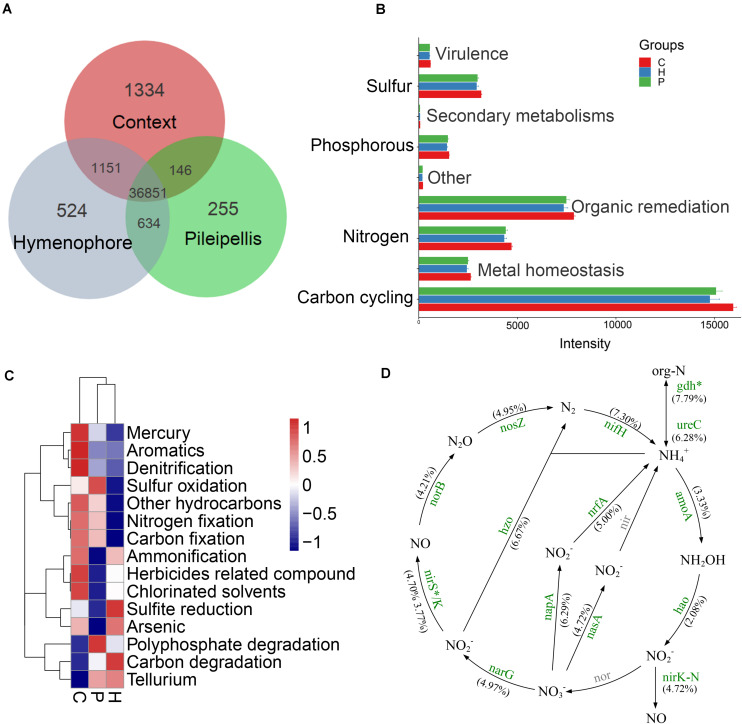
GeoChip analyses of key functional genes of microbial communities in *Thelephora ganbajun* tissue compartments (C, context; H, hymenophore; P, pileipellis). **(A)** Shared and unique functional genes; **(B)** gene category ANOVA barplots with significant differences (*P* < 0.05); **(C)** top 15 gene subcategory heatmap (colors show mean values, *n* = 3); **(D)** nitrogen cycling analysis from GeoChip data (as no significant differences between H and P, nitrogen cycling comparison was made only between C and H). The percentage change in N-related gene intensity between the context and hymenophore is indicated in parenthesis. Increased gene intensities in the context are labeled in green and significance is marked with *P < 0.05. The gray color genes are not targeted by GeoChip 5.0.

A total of 36,851 fluorescence signal probes were commonly shared across the three compartments of *T. ganbajun* (Figure 4A). The number of unique probes was highest in the context (1,334), followed by the hymenophore (524) and then the pileipellis (255) (Figure 4A). Meanwhile, a total of 364 different functional genes belonging to 8 major gene categories and 42 subcategories were identified (Supplementary Data Sheet 1). Gene intensities involved in C, N, S, and P cycling were significantly higher in the context than in the hymenophore and pileipellis (Figure 4B), but no significant differences between hymenophore and pileipellis were found. The main functional characteristics were largely related to the compartments: the functional genes related to sulfur oxidation and polyphosphate degradation were enriched in the pileipellis; those related to C degradation and sulfite reduction were highest in the hymenophore; and those related to C and N fixation, ammonification and denitrification were enriched in the context (Figure 4C). As all 14 GeoChip-detected functional genes involved in N cycling were similar between the hymenophore and pileipellis, we selectively compared N cycling gene differences between the context and hymenophore. Intensities of all 14 N-related genes were increased in the context compared with the hymenophore (Figure 4D); the highest significant increase (*P* < 0.05) in terms of gene intensity being recorded in the *gdh* gene that plays key roles in N assimilation and N Use Efficiency. Additionally, N-fixation and the reduction of nitrate to nitrite (the latter being the first step for denitrification) also increased in the context compared with the hymenophore, as indicated by a higher intensity of both *nifH* and *napA* genes (Figure 4D).

**FIGURE 5:**
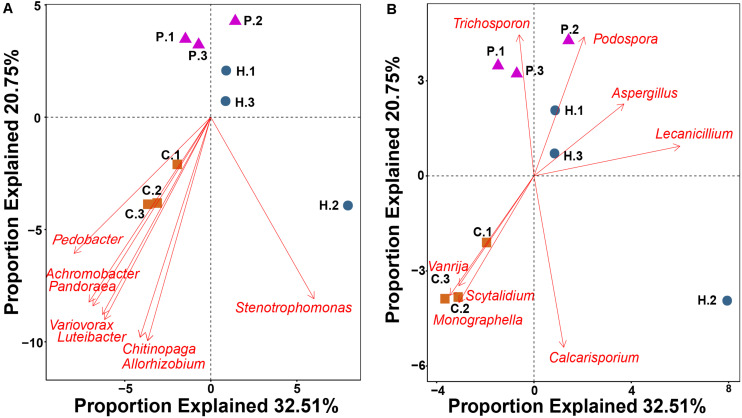
RDA showing the relationship between 16 bacterial **(A)** and fungal **(B)** genera with a highly significant correlation (*P* < 0.01) with functional gene structure within *Thelephora ganbajun* tissue compartments (C, context; H, hymenophore; P, pileipellis). The top 60 genera from amplicon data in the three compartments were used as vectors in the redundancy analysis.

RDA using the top 60 abundant microbial genera as vectors highlighted that 16 genera were significantly correlated (*P* < 0.01) with functional gene structure within the three *T. ganbajun* compartments (Figure 5). Six bacterial (*Achromobacter, Chitinophaga, Luteibacter, Pandoraea, Pedobacter* and *Variovorax*) (Figure 5A) and 3 fungal (*Monographella Scytalidium* and *Vanrija*) genera (Figure 5B) were correlated with the functional gene structure in the context. Meanwhile, the fungal genera *Aspergillus* and *Lecanicillium* associated with functional genes in the hymenophore, while *Trichosporon* significantly (*P* < 0.01) associated with functional genes in the pileipellis (Figure 5B).

## Discussion

### Microbial Diversity and Structure Differs Between Compartments

In line with our first hypothesis (H_1_) a clear compositional dissimilarity among *T. ganbajun* compartments was recorded (Figure 3). This indicates that the three studied compartments represent distinct niches, each harboring a different microbiome community structure. The clear beta-diversity shift (Figure 3) indicates that the sporocarps are composed of complex microniches. *T. ganbajun’*s pileipellis harbored higher bacterial diversity and functional genes than those in the hymenophore (Figures 2, 4), indicating distinct bacterial partitioning in the micro-niches of the outer layer of the fruiting body where the basidiospores are borne. Similarly, a higher bacterial diversity in pileus (compared to stipe) was identified in the edible mushroom of *Morchella sextelata* M. Kuo (Benucci et al., 2019). The highest bacterial diversity was located in the context—a more internal sterile tissue in *T. ganbajun*’s fruiting body (Figure 2). It is possible that the *T. ganbajun* context is a key aromatically dense niche that recruits bacteria, which can be supported by its aromatic-related functional gene enrichment (Figure 4C) and the close association between EMF and bacterial communities in aroma formation (Piloni et al., 2005; Splivallo et al., 2015; Vahdatzadeh et al., 2015). Additionally, the context’s thick inner layer constitutes a unique micro-environment for C and N fixation (Figure 4C), thus providing energy and nutrition for functional microbes (as indicated by its highest functional gene numbers; Figure 2A). At the Kingdom level, although predominantly populated by bacteria, *T. ganbajun* was also colonized by other filamentous fungi from surrounding soils. As expected, *T. ganbajun*-inhabiting fungal communities had low abundance and there were no significant differences between the three studied compartments (Supplementary Table 1), indicating low occupancy of soil fungi in *T. ganbajun* sporocarps and loose associations across compartments. Similar weak correlations have previously been recorded for other Ascomycota EMF species belonging to the genus *Tuber* (Pacioni et al., 2007; Rivera et al., 2010).

As a metagenomic technique, amplicon sequencing offers unique insights into microbiomes that would be impossible to achieve through culture-based methods. The high sensitivity of amplicon sequencing to assess the microbiome is also a clear advantage over traditional culturing methods (Gupta et al., 2019). Nevertheless, as with all PCR and sequencing methodologies, amplification and coverage biases must be acknowledged. Limitations of amplicon sequencing remain (i) the choice of the primer pair for amplification which leads to variations in defining the accuracy of microbial profiles (Mancabelli et al., 2020); and (ii) PCR amplification biases that hinder accurate representation of microbial population structures and interpretation of relative abundances from amplicons (Cottier et al., 2018). However, with an awareness of these caveats and by minimizing bias in experimental design and analysis, techniques using 16S hypervariable regions (as performed in our analyses) can confidently assess bacterial community dynamics (Ibarbalz et al., 2014).

### Potential Active Functions in *T. ganbajun* Compartments

In line with our second (H_2_) and third hypothesis (H_3_) related to micro-niche functional gene structure and variation in signal intensities, the uniqueness of compartment-related probes/signal intensity (detecting functional genes, multiply probes for individual genes) was almost twice as high in the context (1,334) compared to the total of the two other compartments (779; Figure 4A). The significantly distinct functional genes within compartments were compared based on their different gene signal intensities (Supplementary Image 1). Genes showing the highest differences were those relating to C fixation (*mdh, 4hbcd, 4hbcd_dic4hb*), C degradation (*apu*), organic remediation (*phtb, chnc*), metal homeostasis (*merg, chrr*), and aromatic (*nfsb_2*) (Supplementary Image 1).

Genes across the three compartments in *T. ganbajun* were investigated further. Among a total of 364 genes, 352 genes (96.7%) were recorded in all compartments (Supplementary Data Sheet 1). Of these, the most abundant genes relevant to forest ecosystem functioning included those for C fixation (*CsoS1_CcmK, FBPase, TIM*), N fixation (*nifH*), P utilization (*ppx, ppk*), and S metabolism (*dsrB*, *dsra*) (Supplementary Data Sheet 2). *T. ganbajun* could, therefore, drive carbon-fixation and biogeochemical element cycling through its microbiome. From the perspective of an ecological niche within an individual fungus, the major functional role of *T. ganbajun* might be tissue compartmentation dependent (H_3_). In agreement with H_3_, the context could thus be a functional hotspot contributing significant to biogeochemical cycling (Figure 4B).

Interestingly, the pileipellis was enriched with functional genes for sulfur oxidation, which could indicate the presence of aggregating autotrophic S-oxidizing bacteria that utilize S as an energy source (Norris et al., 2011; Wang et al., 2019). Thirty-three bacterial genera harboring S-oxidizing genes (*SoxA*, *SoxB*, *SoxC*, and *SoxY*) were enriched in the pileipellis (Supplementary Data Sheet 2). S-oxidation has previously been conclusively demonstrated in some of these genera, e.g., *Acidithiobacillus* (Thurston et al., 2010), *Chlorobium* (Eisen et al., 2002), *Chlorobaculum* (Keppen et al., 2008), *Halothiobacillus* (Fike et al., 2016), and *Methylobacterium* (Anandham et al., 2008). The hymenophore could also be the site of sulfite-to-sulfide reduction, demonstrating its essential role in the assimilatory sulfur reduction pathway (Canfield, 1999; Canfield et al., 2005). This was further supported by a higher sulfite reductase (*Sir*) abundance in the hymenophore (Supplementary Data Sheet 2). Using the massive functional gene coverage of the GeoChip, results from both the pileipellis and hymenophore could improve our understanding of sulfur-metabolic processes in *T. ganbajun*.

N fixation-associated gene intensity was highest in the context compartment (Figure 4C). Usually N fixation needs a *sine qua non-environment*: a reduction of oxygen concentration and anaerobiosis. Numerous nitrogen-fixing organisms exist only under an anaerobic condition, which was in the context but not in the pileipellis and hymenophore niches (Supplementary Data Sheet 2). As a common biomarker for N-fixing bacteria, *nifH* gene diversity was highly enriched in the context (Supplementary Image 2). This gene was also detected in a range of microbial taxa including the Archaea phylum Euryarchaeota and some bacterial phyla including Actinobacteria, Chlorobi, Chloroflexi, Cyanobacteria, Firmicutes, Spirochetes, Proteobacteria, and Verrucomicrobia (Supplementary Data Sheet 1).

We further explored all functional genes involved in N cycling. Given that N-related gene signal intensities were similar between the hymenophore and pileipellis in *T. ganbajun*, N cycle analyses were selectively compared between the context and hymenophore. The high abundance of both *nirK* and *amoA* genes suggests that nitrification and denitrification processes are simultaneously boosted in the context (Figure 4D). In addition, the context’s greater abundance of *gdh* genes, involved in N mineralization, denotes the presence of ammonifying bacteria (e.g., *Bacillus, Clostridium, Micrococcus, Pseudomonas*, and *Sharaea*) and archaea (*Halobacterium, Haloferax, Methanosaeta, Thermococcus*, *Thermoplasma*, and *Thermoproteus*) (Supplementary Data Sheet 1). All of these contributors play potential roles in decomposing organic nitrogen-containing compounds to produce ammonia (Sepers, 1981; Rusch, 2013). Furthermore, this microbiome suggests that the context’s anoxic environment is a major driver of this compartment’s microbial composition, and a key contributor to the *T. ganbajun* sporocarp’s chemical profile.

## Conclusion

This study presents for the first time comprehensive insights into the structure and potential functions of the microbiome in different tissue compartments of a Basidiomycota mushroom, *T. ganbajun*, based on a polyphasic approach of a functional gene microarray (GeoChip) and amplicon sequencing. Our GeoChip results demonstrate that the *T. ganbajun’*s context compartment provides a favorable habitat to microorganisms with a range of genes of great functional importance. The detected functional genes mainly associated with C, N, P, and S cycling, as well as remediation of organic materials. These results provide novel insights into the differential roles of fungal tissue compartments, as drivers of microbiome composition and functional gene diversity, as well as the ecological and biogeochemical functions of sporocarp-forming fungi in forest ecosystems.

Additionally, this study contributes to the identification of potential symbionts, including “helper bacteria” involved in mushroom development, that may be important to develop a *T. ganbajun* cultivation program. Future transcriptomic, metabolomic and functional studies can be used to analyze the relative importance of these sporocarp-associated microbial communities, thereby identifying commensal organisms from growth-promoting ones, or those involved in enhancing fungal nutraceutical properties. Through further characterization of mushroom-dwelling microbes we may also shed light on the mechanisms underlying fungal mutualisms and the co-evolutionary processes guiding them.

## Data Availability Statement

The datasets presented in this study can be found in online repositories. The names of the repository/repositories and accession number(s) can be found in the article/Supplementary Material.

## Author Contributions

DL prepared the original draft and was responsible for sequencing data analysis. JP-M, XH, and CC improved figures, reviewed, and edited the manuscript. JP-M and FY designed the whole experiment. DL and FY got the funding. All authors contributed to the article and approved the submitted version.

## Conflict of Interest

The authors declare that the research was conducted in the absence of any commercial or financial relationships that could be construed as a potential conflict of interest.

## Footnotes

1 http://www.drive5.com/usearch/
