# Supplementary material for: Microbiome Community Structure and Functional Gene Partitioning in Different Micro-Niches Within a Sporocarp-Forming Fungus
Source: Front Microbiol. 2021 Mar 30;12:629352. doi: 10.3389/fmicb.2021.629352 (PMC8042227; doi:10.3389/fmicb.2021.629352)
Supplement: Supplementary file 3 [file Image_1.pdf]

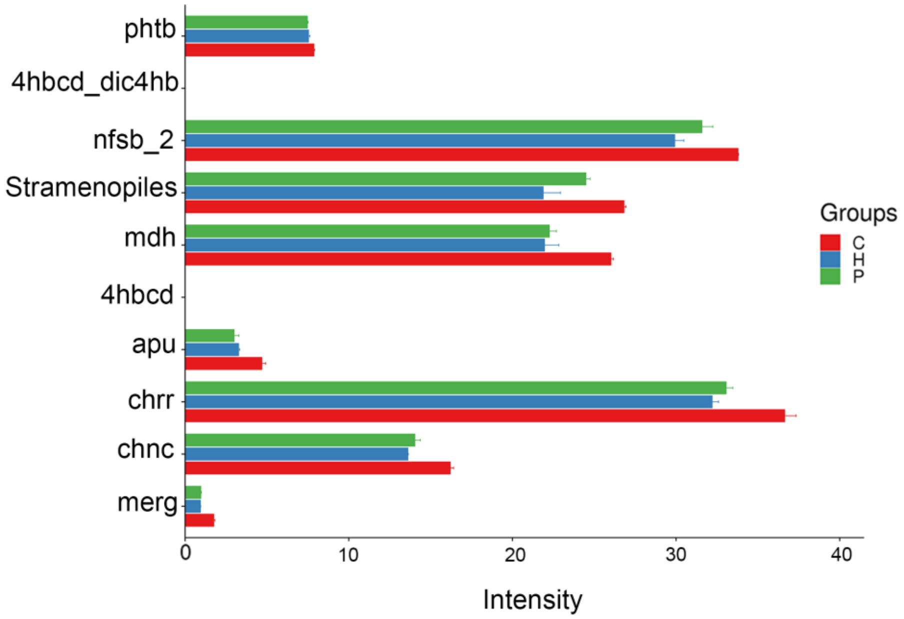

Supplementary Figure 1. Genes with the highest significant differences (P = 0.01) among *Thelephora ganbajun* compartments (C = context, H=hymenophore and P = pileipellis). Specific genes are those relating to C fixation (*mdh*, *4hbcd*, *4hbcd_dic4hb*), C degradation (*apu*), organic remediation (*phtb*, *chnc*), metal homeostasis (*merg*, *chrr*), and aromatic (*nfsb_2*). Values are the mean of the three bio-replicates.
