# Supplementary material for: Microbiome Community Structure and Functional Gene Partitioning in Different Micro-Niches Within a Sporocarp-Forming Fungus
Source: Front Microbiol. 2021 Mar 30;12:629352. doi: 10.3389/fmicb.2021.629352 (PMC8042227; doi:10.3389/fmicb.2021.629352)
Supplement: Supplementary file 4 [file Image_2.pdf]

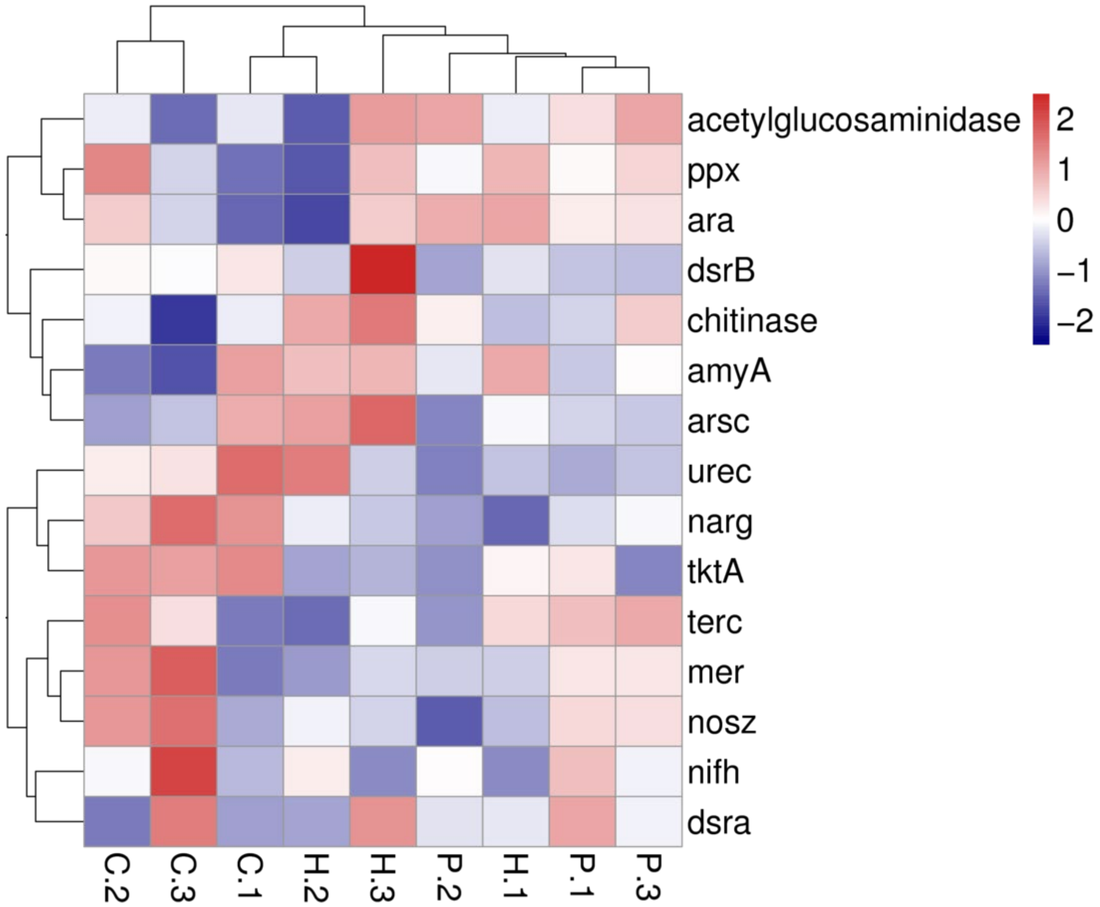

Supplementary Figure 2. Heatmaps of top 15 functional gene intensities of *Thelephora ganbajun*'s three tissue compartments (C = context, H = hymenophore and P = pileipellis). Arabic numerals (1, 2 and 3) indicate three bio-replicates.
