# Supplementary material for: Microbiome Community Structure and Functional Gene Partitioning in Different Micro-Niches Within a Sporocarp-Forming Fungus
Source: Front Microbiol. 2021 Mar 30;12:629352. doi: 10.3389/fmicb.2021.629352 (PMC8042227; doi:10.3389/fmicb.2021.629352)
Supplement: Supplementary file 5 [file Table_1.doc]

| **Table S1.** Alpha diversity changes in filamentous fungal communities of *Thelephora ganbajun’*s three compartments. | | | | | |
| --- | --- | --- | --- | --- | --- |
| Sample ID | Chao1 | Dominance | Observed_species | Shannon | Simpson |
| C.1 | 98.93729 | 0.902243014 | 79 | 0.476251 | 0.097757 |
| C.2 | 90.90591 | 0.961330408 | 55 | 0.212749 | 0.03867 |
| C.3 | 83.19985 | 0.969453928 | 50 | 0.171614 | 0.030546 |
| H.1 | 108.4942 | 0.910548348 | 70 | 0.421816 | 0.089452 |
| H.2 | 93.27611 | 0.954100402 | 52 | 0.238431 | 0.0459 |
| H.3 | 83.73373 | 0.91704528 | 66 | 0.392255 | 0.082955 |
| P.1 | 93.57176 | 0.909858234 | 75 | 0.426921 | 0.090142 |
| P.2 | 95.95455 | 0.907251815 | 80 | 0.445602 | 0.092748 |
| P.3 | 95.84839 | 0.975731608 | 54 | 0.142526 | 0.024268 |

Abbreviations: C = context, H=hymenophore and P=pileipellis. Arabic numerals (1, 2 and 3) following these abbreviations indicate three bio-replicates.
